# Muscle Structure and Function Recovery: Adalimumab‐Calcium Channel Synergy in Post–Ischemic Stroke Sarcopenia

**DOI:** 10.1002/jcsm.70097

**Published:** 2025-11-10

**Authors:** Hu Qi, Xiong‐Wei Zhang, Ze‐Yang Zhang, Shan‐Shan Ou, Yuan‐Lin Gao, Dan Tian, Yan‐Ning Jiang, Xin‐Ran Min, Ao‐Tao Zhao, Jia‐Min Zou, Jiu‐Seng Zeng, Qiu‐Yi Pu, Ruo‐Cong Yang, Nan Zeng

**Affiliations:** ^1^ Lab for Innovation & Effective Uses of Chinese Drug Germplasm Resources Chengdu University of Traditional Chinese Medicine Chengdu Sichuan Province China; ^2^ School of Pharmacy Chengdu University of Traditional Chinese Medicine Chengdu Sichuan Province China; ^3^ Acupuncture and Tuina School Chengdu University of Traditional Chinese Medicine Chengdu Sichuan Province China; ^4^ Department of Traditional Chinese Medicine Chengdu Integrated TCM& Western Medicine Hospital Chengdu China

**Keywords:** adalimumab, Ca^2+^ channels, combination therapy, ischemic stroke, muscle structure

## Abstract

**Background:**

Adalimumab, a TNF‐α inhibitor, is widely used clinically. Recent studies suggest Adalimumab can improve muscle damage after ischemic stroke (IS), but its protective mechanisms remain unclear. This study investigates the effect of adalimumab on muscle structure post‐IS and the role of calcium balance in muscle strength, while validating their synergistic effect.

**Methods:**

This study investigates the effects of adalimumab and GV‐58 on muscle structure and function in male middle cerebral artery occlusion (MCAO) rat models using behavioural, imaging, pathological and WB experiments. In vitro mechanisms are explored with L6 and primary muscle cells.

**Results:**

The results of the present study showed a significant decrease in motor function in IS‐induced sarcopenia (ISS) rats, as evidenced by shortened length (−47.56%, *p* < 0.001), reduced weight (−43.79%, *p* < 0.001) and reduced cross‐sectional area of myofibroblasts (−38.58%, *p* < 0.001) in the soleus muscle as compared to the sham group. Inflammatory factors such as IL‐1β, IL‐6, TNF‐α and reactive oxygen species (ROS) levels were significantly elevated in the muscles of ISS rats (3.10‐fold, 3.78‐fold, 2.29‐fold, 2.80‐fold, *p* < 0.001). Molecular mechanism studies showed that TNF‐α, MAFbx and MuRF1 protein expression was down‐regulated, and IL‐10 and MyoD1 expression was up‐regulated in muscle tissues of ISS rats. RNA‐seq implicated the Ca^2+^ signalling pathway in ISS‐related muscle weakness. Muscle strength in ISS rats is associated with Ca^2+^ content and Ca^2+^ channels, and key excitation–contraction coupling proteins SERCA2, Cav1.1 and RYR1 expression was decreased, whereas Ca^2+^ sensing proteins STIM1 and CAM expression were compensatory upregulated. Adalimumab treatment significantly reduced muscle inflammation and structural damage in ISS rats, significantly increasing the length (+66.88%, *p* < 0.001) and weight (+43.92%, *p* < 0.001) of the soleus muscle and increasing muscle cell cross‐sectional area (+53.44%, *p* < 0.001). Adalimumab also inhibited the expression of MAFbx, MuRF1 and promoted the expression of IL‐10 and MyoD1. GV‐58 treatment of L6 cells showed that combined administration with adalimumab produced a synergistic effect. Upregulation of key Ca^2+^ protein expression such as RyR1 and SERCA1 improved the recovery of muscle strength in ISS rats while maintaining muscle structure.

**Conclusions:**

The combination of adalimumab and GV‐58 effectively restores muscle function after stroke by inhibiting inflammation and improving calcium channel dysfunction.

## 1 Introduction

Ischemic stroke (IS) is one of the leading lethal diseases worldwide and the leading cause of long‐term disability; approximately 80% of the survivors experience motor dysfunction [1]. The ISS is a common occurrence affecting about 60%–75% of patients and is primarily characterized by a decline in skeletal muscle mass and strength [2].

The pathological mechanisms of ISS involve neuroplasticity, inflammatory response, imbalance of protein metabolism and mitochondrial dysfunction [3]. The key drivers of ISS include TNF‐α–mediated systemic inflammation and oxidative stress [4]. Advancements have been made in optimising ischaemia–reperfusion and neuroprotective strategies during the acute phase of IS. However, the specific pharmacological interventions for functional limb recovery during the IS rehabilitation phase are still lacking [5]. Existing rehabilitation approaches inadequately reverse muscle fibre atrophy and strength loss, and studies show this damage is largely irreversible [6].

Adalimumab, a monoclonal antibody that specifically targets TNF‐α, serves as the first‐line treatment for ankylosing spondylitis (AS) and can effectively alleviate skeletal pain and stiffness while improving functional mobility in patients [7]. Numerous studies have demonstrated that adalimumab exhibits significant therapeutic efficacy in various inflammatory diseases [8, 9, 10]. It can target TNF‐α [11] and block its binding to TNFR1/2 receptors, thereby inhibiting the activation of downstream inflammatory signalling pathways, such as NF‐κB and MAPK. Studies have shown that the NF‐κB and MAPK pathways [11], which are the key upstream regulators of the ubiquitin‐proteasome system, can modulate the expression of E3 ubiquitin ligases MuRF1 and MAFbx, thereby directly affecting the degradation and synthesis of muscle proteins [12]. Furthermore, the excess of TNF‐α can inhibit the differentiation capacity of muscle satellite cells and suppress the Akt/mTOR/MyoD1 signalling pathway that is involved in muscle protein synthesis [13]. L6 rat skeletal myoblast cells stably express mature skeletal muscle proteins and signalling pathways, providing an ideal model for muscle atrophy research. GV‐58 is a potent and highly selective agonist for the N‐type (Cav2.2) calcium channels [14], demonstrating superior subtype specificity for Cav2.2 over other voltage‐gated calcium channels [15].

Previous studies showed that inflammatory factors, including TNF‐α, IL‐6 and IL‐1β, were significantly upregulated in the soleus muscles of MCAO rats [4]. The pharmacological intervention simultaneously downregulated these inflammatory factors and reversed muscle atrophy and contractile dysfunction [16]. The current study focused on the pivotal role of TNF‐α in mediating the ISS pathology and its global regulation of the balance between muscle protein synthesis and degradation. The multidimensional mechanisms by which TNF‐α could mediate the restoration of muscle mass and motor function in sarcopenia were systematically explored by integrating molecular regulatory networks and functional phenotypic analysis.

This study aimed to elucidate the therapeutic potential of adalimumab in targeting and inhibiting the TNF‐α signalling pathway to improve poststroke imbalance of muscle protein metabolism as well as regenerative dysfunction. This study not only presented the first targeted biologic intervention strategy for ISS but also innovated a cross‐organ regulatory theoretical framework for central nervous system injury and skeletal muscle atrophy after stroke.

## 2 Materials and Methods

### 2.1 Model Preparation, Grouping and Drug Treatment

Male Sprague–Dawley rats (weight: 240–260 g) were obtained from SPF Biotechnology (Beijing) Co. Ltd. (No. SCXK‐Beijing‐2024‐001). The rats were housed in an SPF environment at a temperature of 22°C–25°C, relative humidity of 40%–60%, with a 1‐week acclimation period. This protocol was approved by the Laboratory Animal Ethics Committee of Chengdu University of Traditional Chinese Medicine (No. 2024054). A total of 54 rats were included. In part 1, 18 rats were randomly assigned to the following three groups: sham, MCAO and MCAO + adalimumab (4 mg/kg). In part 2, 12 rats were divided into the sham and sham + adalimumab (4 mg/kg) groups. In part 3, 24 rats were allocated into the following four groups: sham, MCAO, GV‐58 (20 mg/kg) and GV‐58 (20 mg/kg) + adalimumab (4 mg/kg). Each group included the following six rats; one outlier was excluded. MCAO modelling was performed under Zoletil 50 following standard procedures [17]. A midline cervical incision was made to expose and isolate the right CCA, ECA and ICA. The CCA and ECA were ligated, and a filament was inserted into the ICA to induce occlusion. In the MCAO model, right‐sided cerebral blood flow was occluded in supine‐positioned rats, leading to contralateral (right‐sided) hemiparesis in the prone position. Adalimumab is administered by intraperitoneal injection at a dose of 4 mg/kg [18] on days 1, 3, 5 and 7. GV‐58 is given by intraperitoneal injection at a dose of 20 mg/kg [15, 19, 20] once daily for 7 consecutive days. The sham and MCAO groups received saline. Please refer to Table S1–3 for all reagents, abbreviations and instrument information.

### 2.2 Primary Cell Preparation and Pretreatment

Rat soleus muscles were dissected, trimmed of fat and connective tissue [21], and rinsed with Hanks' buffer after dual‐antibody treatment. Tissues were minced (~1 mm^3^) and digested at 37°C with 0.1% Collagenase II and 0.25% trypsin (3:1) in two steps (25 and 20 min) [22]. After digestion, the suspension was filtered (100/200 mesh), centrifuged (1000 rpm, 5 min) and resuspended in DMEM with 20% FBS. Cells were seeded on 0.1% gelatin‐coated dishes (5 × 10^4^/cm^2^) and cultured at 37°C, 5% CO₂ [23]. After adherence, a laser microdissection system (Leica, LMD7) was used to remove irregular or aggregated cells, and typical spindle‐shaped myoblasts were selected for further experiments.

### 2.3 Chemicals and Reagents

All reagent and antibody details can be found in Table S1.

### 2.4 Laser Spot Detection

Cerebral perfusion on the infarcted side was assessed using a laser speckle imaging system. The skulls were irradiated with a 671‐nm laser, and images were captured at 50 fps with a RWD (RFSLI ZW/RFLSI III) device. Images were analyzed with RWD perfusion software to calculate the mean region of interest (ROI).

### 2.5 Neurological Deficit Tests (Zea‐Longa and Bederson Score)

Neurological deficits were assessed on days 1 and 7 after MCAO using the Zea‐Longa double‐blind test and Bederson scoring system, with Zea‐Longa scores ranging from 0 (*no injury*) to 4 (*no response to stimuli*), 0: *Asymptomatic or mild neurological dysfunction*, 1: *Hemiplegia*, 2: *Moderate hemiplegia, but able to walk*, 3: *Severe hemiplegia, unable to walk* and 4: *Loss of brainstem function and being in a vegetative state* [24]. Bederson scoring system ranges: 0: *No deficit*, 1: *Forelimb flexion when lifted by tail*, 2: *Reduced resistance to lateral push (left/right)* and 3: *Spontaneous circling behaviour* [25] .

### 2.6 Brain TTC Staining

Brain tissues were collected, soaked in PBS for 5 min, frozen at −20°C for 20 min, and sectioned at 2 mm. Brain slices were incubated in 1% TTC at 37°C for 10 min and then fixed in 4% paraformaldehyde for 24 h. Photographs were taken to record and calculate the cerebral infarct area [26].

### 2.7 Tolerance and Capability of Exercise

Rat body weights were measured on days 1, 3, 5 and 7 after MCAO. Endurance and locomotor function were assessed on a treadmill (SANS, SA101B) with a 1.50 mA current and 10 m/min speed. Maximum pulling force was measured using the SA417 Grasping Force Tester (Jiangsu Zhongke Biotechnology Co. Ltd.), repeated three times, and averaged for analysis [27].

### 2.8 Soleus Muscle Length and Weight

On day 7 after MCAO, the right soleus muscle of rats was dissected. The length and weight of all samples were measured and recorded.

### 2.9 H&E Staining of Soleus

Rat soleus muscle was fixed in 4% paraformaldehyde, dehydrated, embedded and sectioned. Sections were deparaffinized, rehydrated and stained with haematoxylin for 8 min and eosin for 2 min. After sealing with neutral resin, sections were observed under a microscope to analyze muscle cross‐sections and inflammatory infiltration.

### 2.10 Detection of IL‐6, TNF‐α and IL‐1β Levels in Soleus

Twenty milligrams of soleus were homogenized and lysed using collagenase II and then centrifuged at 5000 g for 10 min at 4°C, and the supernatant was collected to determine the levels of IL‐6, TNF‐α and IL‐1β factors in rat soleus muscle by ELISA (Jiangsu Meimian Industrial Co. Ltd).

### 2.11 Detection of ROS by Flow Cytometry

Fresh soleus muscle was dissected, then cut with scissors and digested with type II collagenase to single‐cell suspensions, and ROS levels in halibut muscle were assessed using a ROS detection kit and analyzed by flow cytometry (BD, FACSCanto II).

### 2.12 Muscle Electrical Signal Acquisition

The muscle electrical signals of the right soleus muscle of rats were detected using a physiological electrical signalling system (Chengdu Instrument Factory, Cat: RM6240XC). The electrodes were inserted into the right side of the muscle at an angle of 45°, and the peak maps of the muscle electrical signals were recorded, and the peaks were statistically analyzed [27].

### 2.13 Muscle Ultrasound Testing

Muscle thickness was measured using a high‐resolution small animal ultrasound system on days 1 and 7 after MCAO. After isoflurane anaesthesia, rats were positioned laterally, and the right muscle thickness was measured with the probe at a 45° angle. Image data were acquired and analyzed using Vevo analysis software [28].

### 2.14 Transmission electron Microscopy

Fresh soleus muscle was fixed with 3% glutaraldehyde and 1% osmium tetroxide, then embedded in Epon‐812. Thin sections (70 nm) were stained with 3% uranyl acetate, washed, then stained with 2.7% lead citrate and washed again. The sections were observed and photographed using a transmission electron microscope (JEOL, Japan).

### 2.15 Immunohistochemical Detection of in Soleus Muscle (IHC)

Paraffin sections of rat soleus muscle were deparaffinized, antigen‐retrieved, blocked, and sealed. After overnight incubation with primary antibodies (TNF‐α, MuRF1, MAFbx, Cav1.1 and RyR1) at 4°C, sections were incubated with secondary antibodies for 1.5 h at room temperature. Positive staining was visualized with DAB, and nuclei were stained. Images were captured with a panoramic scanning system, showing blue nuclei and brown positive staining.

### 2.16 Cell Culture

L6 cells were obtained from the Cell Bank of the Cell Resource Center of the Chinese Academy of Sciences (Shanghai). Cells were cultured in DMEM medium (Gibco) supplemented with 10% fetal bovine serum (FBS, Gibco) and incubated at 37°C in a humidified atmosphere of 5% CO_2_ and 95% air. In vitro experiments, GV‐58 was treated at a dose of 15 μM and adalimumab at a dose of 10 μg/mL [29, 30, 31]. For the cytotoxicity assay, refer to the Cell Counting Kit‐8 instructions.

### 2.17 Immunofluorescence Analysis

Cells were fixed with 4% paraformaldehyde, permeabilized with 0.5% Triton X‐100, and blocked with 10% BSA. After washing, cells were incubated overnight with primary antibodies against MyoD1 and Phalloidin. After secondary antibody incubation, samples were observed under a super‐resolution microscope (Leica, TSC SP8 STED).

### 2.18 Co‐Immunoprecipitation

L6 cells (fourth generation) were seeded in 100 mm dishes and cultured in complete medium. Upon confluence, cells were lysed with IP buffer, centrifuged, and the supernatant divided. One aliquot was incubated with antibodies against TNF‐α, MuRF1 and MAFbx, while the other was denatured with upwelling buffer as the input control. IP samples were incubated overnight at 4°C, followed by a 12‐h incubation with protein A/G PLUS‐Agarose beads. Immunoprecipitated complexes were analyzed by Western blotting (WB).

### 2.19 Cellular Thermal Shift Assay

Cells treated with adalimumab and controls were resuspended in PBS with protease and phosphatase inhibitors, lysed by heating (37°C–79°C) and freeze–thaw cycles. Lysates were centrifuged at 12000 g for 20 min at 4°C. Equal volume of 2 × SDS buffer was added, boiled, and 20 μL loaded for WB.

### 2.20 mRNA Sequencing and Analysis Methods

Total RNA was extracted from soleus muscle using the RNA Mini Kit. mRNA sequencing was performed by Novogene Co. Ltd. (Beijing) using the Quarseq RNA Library‐Single‐S protocol. Sequencing was performed on the Illumina PE150 platform. Differential expression was analyzed using DESeq2 (R/Bioconductor) with|log2 fold change| > 1 and *p* < 0.05. Gene annotation and enrichment were performed using DAVID and visualized with TBtools.

### 2.21 Alizarin Red S Staining

Soleus muscle sections were deparaffinized in xylene, rehydrated through graded ethanol, washed with PBS, and fixed in 95% ethanol for 10 min. Sections were stained with 2% Alizarin Red S (pH = 4.2) for 10 min, mounted on coverslips, and examined for orange‐red calcium deposits.

### 2.22 Flow Cytometry for Detecting Ca^2+^ Content

L6 cells (1 × 10^5^ cells/mL) were seeded in six‐well plates. Refer to the previous description for cell extraction and pretreatment of primary cells. After drug treatment, the cells were rinsed with PBS, collected, and incubated with Fluo‐4 AM for 30 min at 37°C. The cells were washed with PBS and analyzed by flow cytometry (BD, FACSCanto II).

### 2.23 Western Blot Analysis

Western blot analysis (WB) was performed on soleus muscle tissue and primary cells. After sample lysis, protein concentration adjustment, electrophoresis, membrane transfer and blocking, the cells were incubated overnight at 4°C with primary antibodies for TNF‐α, MuRF1, MAFbx, Myostatin, MyoD1, IL‐10, Cav1.1, STIM1, CaM, SERCA2, Troponin I, CASQ1, Calpain 1, RyR1, TNNC2, Annexin A2,β‐Tubulin, Vinculin and GPADH. Please refer to Table S1 for detailed information on antibodies. Incubate with HRP‐conjugated secondary antibody for 1.5 h at room temperature. The BCA quantitative KIT (Kit‐BCA01) was purchased from Sino Biological Inc. Protein bands were visualized with an ECL kit and captured with a ChemiDoc system (Bio‐Rad, USA). Bands were quantified and analyzed by using Image Lab 3.0 SPSS.

### 2.24 Ethics Statement

All Institutional and National Guidelines for the care and use of animals were followed. The experiment has been reviewed by the ethics committee of Chengdu University of Traditional Chinese Medicine, and the batch number is 2024054.

### 2.25 Statistical Analysis

Statistical analysis was performed using SPSS software. Data are shown as mean ± SD. If the data met the assumption of normal distribution, univariate analysis of variance (ANOVA) and the Tukey test were used to analyze the differences among the groups. In instances where the data did not adhere to a normal distribution, the nonparametric Kruskal–Wallis test was applied. *p* < 0.05 was considered statistically significant.

## 3 Results

### 3.1 Adalimumab Preserves Skeletal Muscle Integrity Without Improving Poststroke Motor Function

**FIGURE 1:**
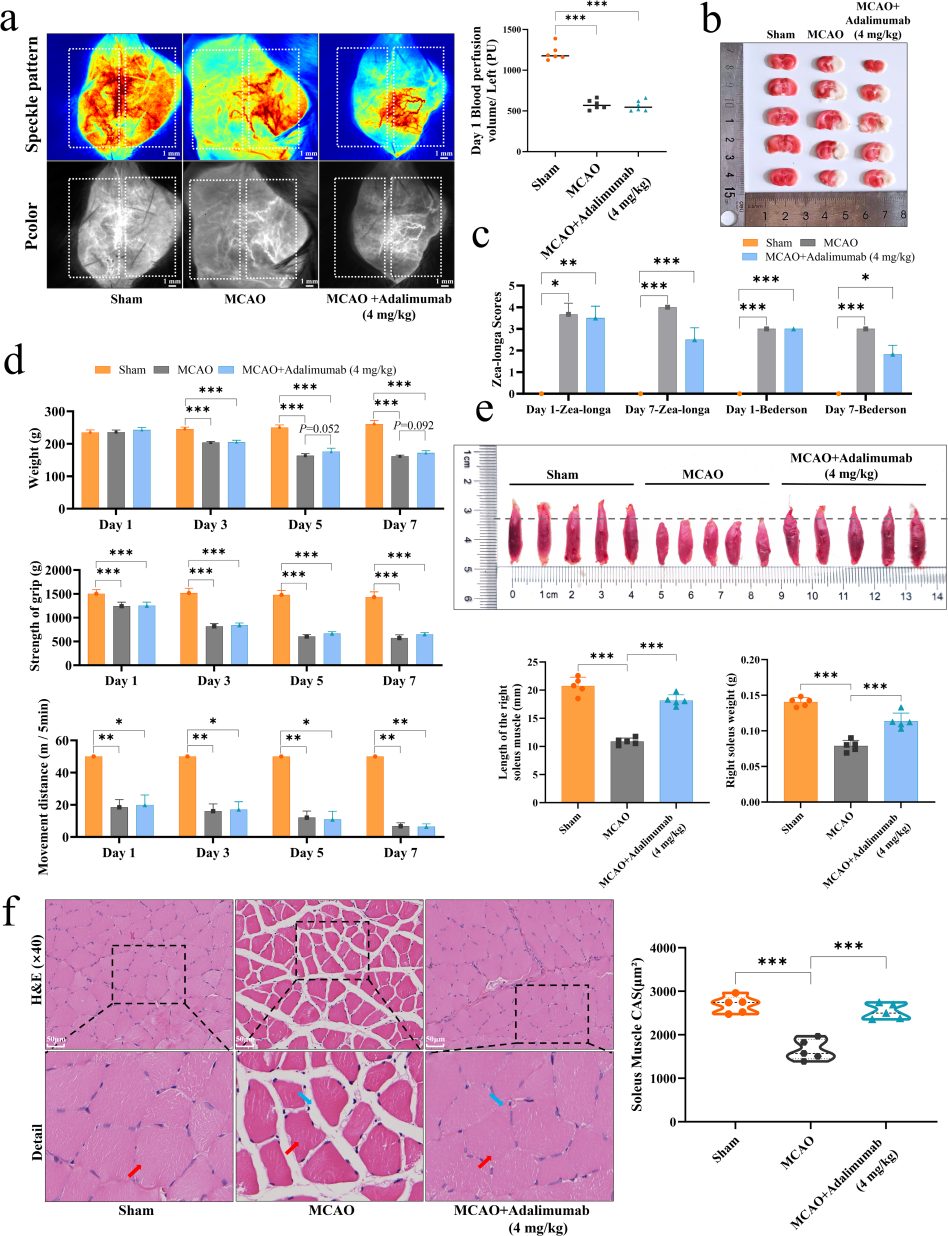
Characterization of ischemic injury and skeletal muscle dysfunction in MCAO rats. (a) Representative images of laser speckle in rat brains and analysis of blood flow perfusion on days 1, *n* = 6. (b) Triphenyltetrazolium chloride staining of brain sections on day 7, *n* = 5. (c) Zea‐Longa neurological score and bederson score assessment of motor impairment. (d) Analysis of body weight, forelimb grip strength, and movement distance in rats. (e) Images of soleus muscle from each group and quantification of soleus muscle length and weight, *n* = 5. (f) H&E staining of soleus muscle showing morphological alterations, scale bar = 50 μm, *n* = 5. *p < 0.05, **p < 0.01, ***p < 0.001.

After confirming no significant differences in central perfusion among surgical groups using laser speckle imaging (Figure 1a), adalimumab intervention was applied. TTC staining, Zea‐Longa scores, and Bederson scores indicated that adalimumab did not improve perfusion in the infarcted hemisphere or neurological function (Figure 1b–c). From day 3 after surgery, adalimumab failed to improve MCAO‐induced declines in body weight, grip strength and locomotion (Figure 1d). On day 7 after surgery, soleus muscle length and weight were markedly reduced in MCAO rats, but significantly restored following adalimumab treatment (Figure 1e). H&E staining showed smaller muscle fibre cross‐sectional areas (red arrow) and larger intercellular spaces (blue arrow) in MCAO rats compared with sham. Adalimumab reduced intercellular spaces and increased cross‐sectional area (Figure 1f). To exclude the effect of adalimumab on healthy rats, we treated sham rats with adalimumab. No significant differences were found for adalimumab on muscle electrical signals, muscle structure and function in healthy rats (ns), as shown in Figure S1a–e. In conclusion, although adalimumab (4 mg/kg) had limited effects on neurologic recovery and overall exercise capacity, it significantly improved the structural integrity of skeletal muscle after MCAO.

### 3.2 Structural Preservation of Muscle Tissue by Adalimumab After Stroke

**FIGURE 2:**
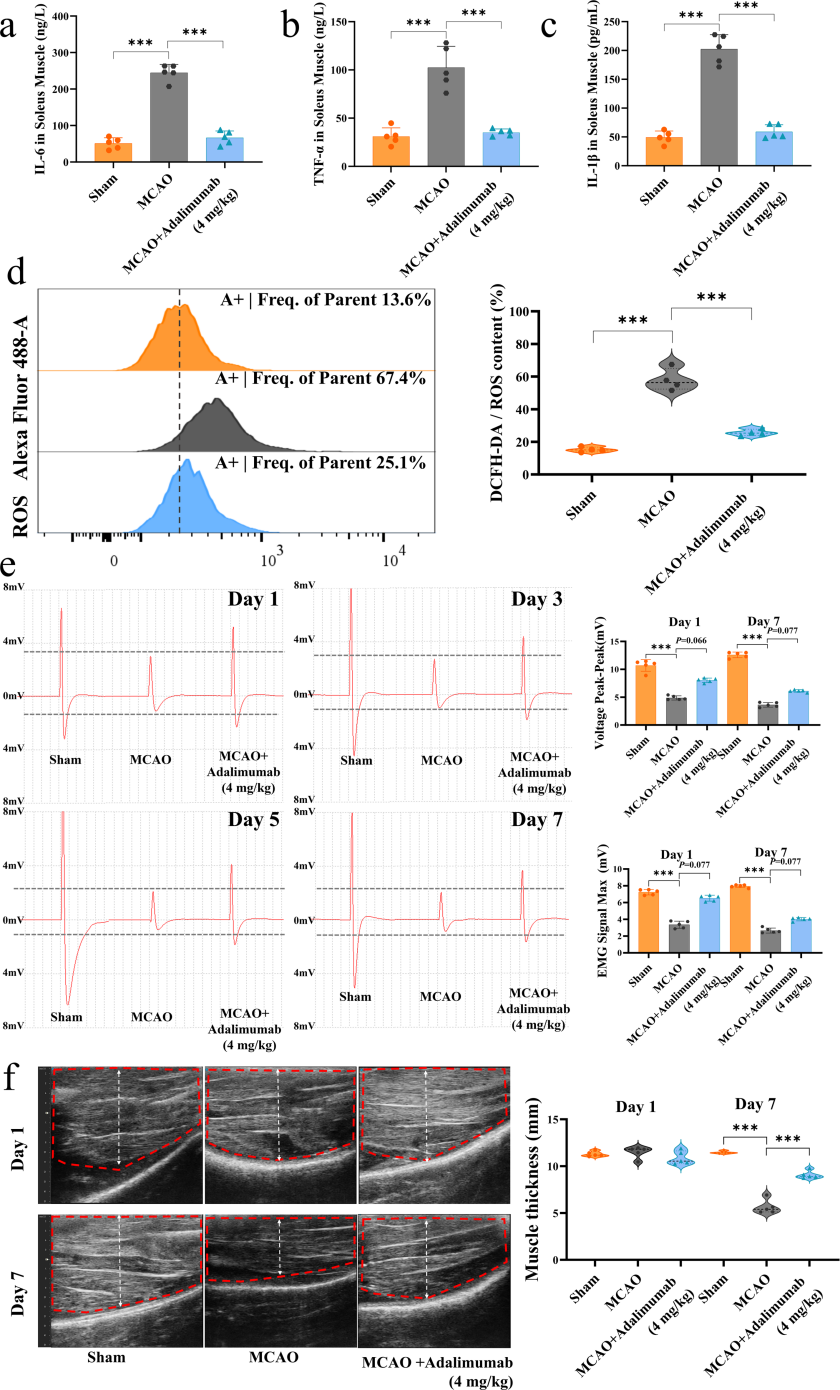
Adalimumab mitigates inflammation and muscle loss in MCAO rats. (a–c) Quantification of proinflammatory cytokines IL‐6, IL‐1β, and TNF‐α in the soleus muscle, *n* = 5. (d) Flow cytometry analysis of reactive oxygen species (ROS) levels in muscle tissue after MCAO, *n* = 4. (e) Representative images of muscle electrical signals in rats and intensity analysis, *n* = 5. (f) Representative ultrasound imaging of muscle morphology and thickness analysis, *n* = 5. *p < 0.05, **p < 0.01, ***p < 0.001.

Adalimumab significantly decreased the levels of inflammatory cytokines IL‐6, TNF‐α and IL‐1β (Figure 2a–c), flow cytometry analysis showed reduced ROS levels in the soleus muscle of MCAO rats (Figure 2d). To evaluate adalimumab's impact on muscle function in MCAO rats, we measured muscle electrical signal intensity after surgery. Compared to the sham group, both the MCAO and adalimumab groups exhibited a significant decrease in peak and maximum muscle electrical signals starting from day 1 after surgery, with no spontaneous recovery observed by day 7 (Figure 2e). Compared to the MCAO group, the adalimumab group showed a trend toward improvement, but no significant difference. Interestingly, muscle ultrasound results indicated that adalimumab significantly improved after MCAO muscle wasting (Figure 2f).

### 3.3 Direct Interaction Between TNF‐α and Muscle Atrophy Markers Mediates Therapeutic Effects of Adalimumab

**FIGURE 3:**
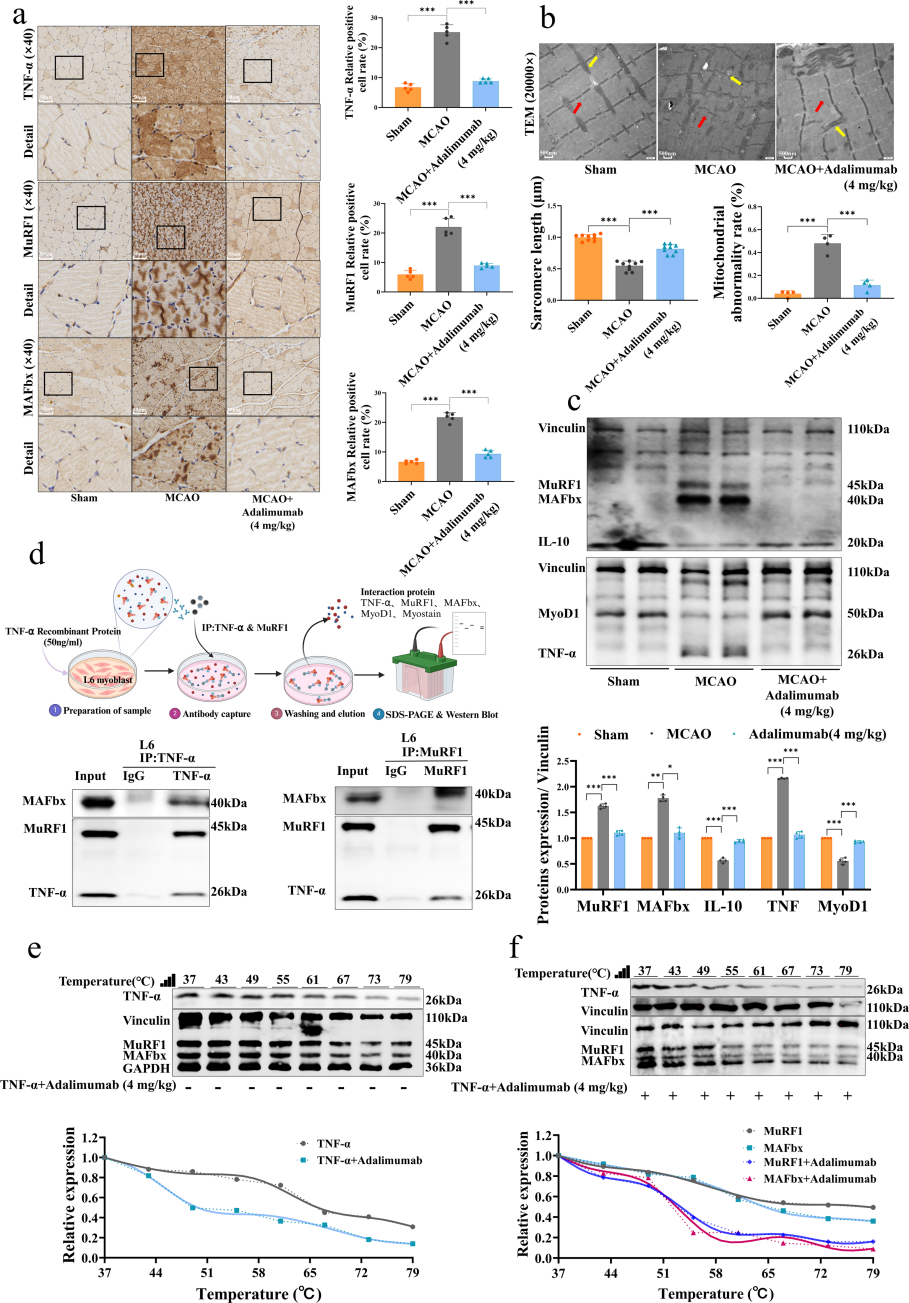
Effects of adalimumab on muscle electrophysiology, ultrastructure, and molecular regulation in MCAO rats. (a) IHC staining and quantification of TNF‐α, MuRF1 and MAFbx expression in the soleus muscle, scale bar = 50 μm. (b) Transmission electron microscopy images of the soleus muscle and corresponding ultrastructural scale bar = 500 nm. (c) WB and relative expression analysis of TNF‐α, IL‐10, MyoD1, MuRF1 and MAFbx in the soleus muscle, *n* = 4. (d) Co‐IP analysis confirming molecular interactions among TNF‐α, MuRF1 and MAFbx. (e‐f) CETSA evaluating the effect of adalimumab on the thermal stability of TNF‐α, MuRF1 and MAFbx. *p < 0.05, **p < 0.01, ***p < 0.001.

Further IHC and WB results indicated that adalimumab significantly downregulated the levels of TNF‐α, MAFbx and MuRF1 in the muscles of MCAO rats, while upregulating the expression of IL‐10 and MyoD1 (Figure 3a). Meanwhile, transmission electron microscopy showed that adalimumab significantly restored myofibril structure and reduced mitochondrial abnormalities after MCAO (Figure 3b–c). This suggests that adalimumab improves muscle atrophy by suppressing tissue inflammation and regulating the balance between muscle protein degradation and synthesis. To investigate the relationship between TNF‐α and muscle atrophy factors MAFbx and MuRF1, we performed co‐immunoprecipitation (Co‐IP) experiments, which revealed an interaction between TNF‐α and both MAFbx and MuRF1 (Figure 3d). Additionally, cellular thermal shift assay (CETSA) results showed that after adalimumab treatment, the thermal stability of TNF‐α, MuRF1 and MAFbx was significantly reduced (Figure 3e–f), suggesting that adalimumab inhibits muscle protein degradation by accelerating the degradation of these proteins.

### 3.4 Ca^2+^ Homeostasis Imbalance as a Central Mechanism of Muscle Dysfunction After Ischemic Stroke

**FIGURE 4:**
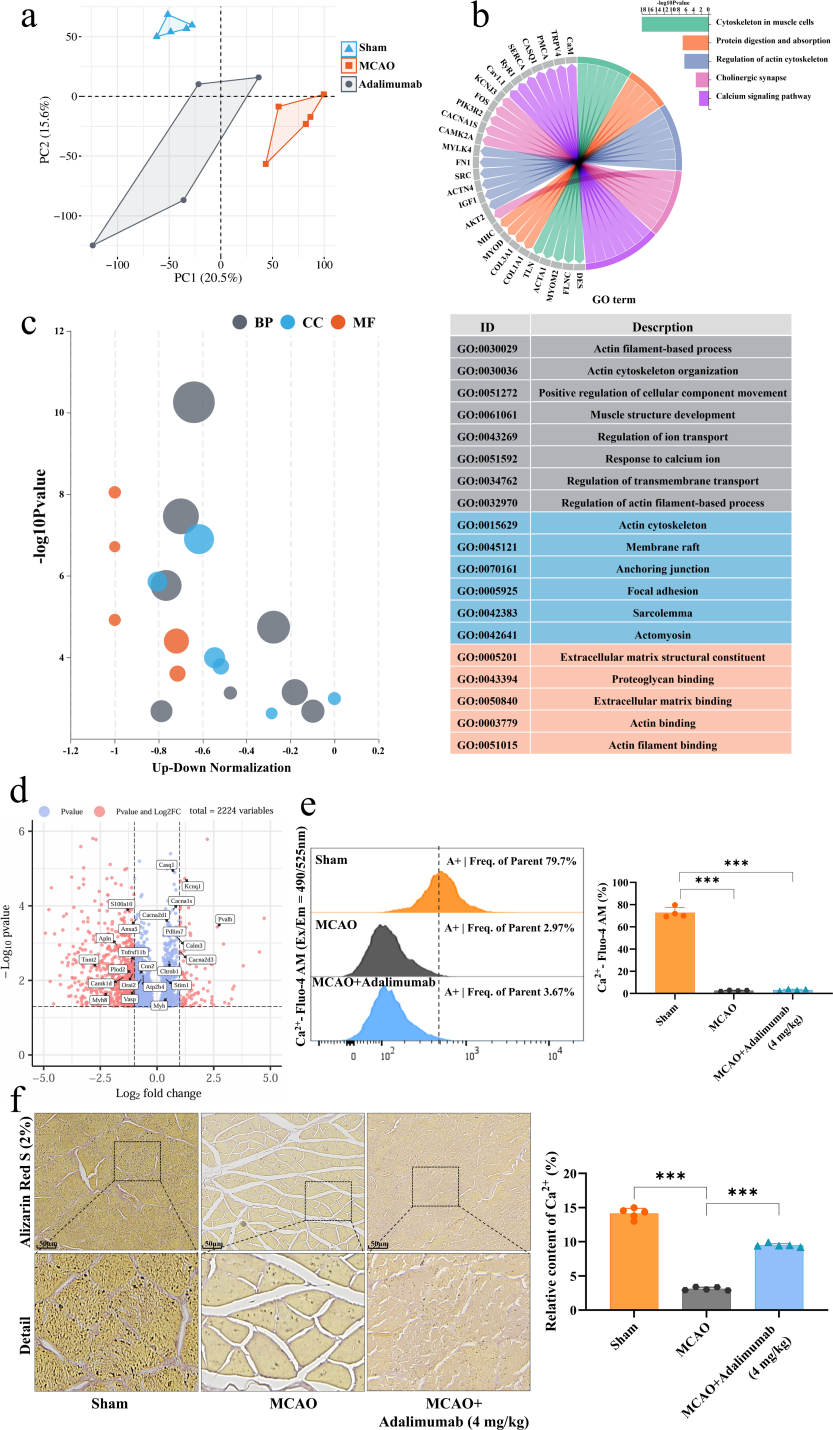
Transcriptomic analysis and calcium signalling‐related molecular changes in the soleus muscle of MCAO rats. (a) Principal component analysis demonstrating sample clustering and variation between experimental groups. (b) KEGG pathway enrichment analysis highlighting key signalling pathways associated with DEGs. (c) Gene ontology analysis illustrating major altered biological processes. (d) Volcano plot depicting the distribution of upregulated and downregulated DEGs. (e) FC analysis of intracellular Ca^2+^ levels in soleus muscle tissue of MCAO rats, *n* = 4. (f) Representative images of Alizarin Red staining of the soleus muscle, scale bar = 50 μm. *p < 0.05, **p < 0.01, ***p < 0.001. DEGs, differentially expressed genes.

To investigate why adalimumab improved muscle structure but not function in MCAO rats, we performed RNA‐seq of soleus muscle. The results showed good sample dispersion across groups (Figure 4a), with key pathways primarily enriched in protein synthesis, Ca^2+^ channels and calcium‐related reactions (Figure 4b). Enriched processes included ion channel function, Ca^2+^ transport, transmembrane transport, actin processing and muscle structural development (Figure 4c), with MYH, CAM, CASQ1 and Cacna1s as key targets (Figure 4d). These results suggest that muscle strength restoration depends on Ca^2+^ content and channel function. To validate the RNA‐seq findings, we assessed Ca^2+^ levels in the rat muscles after surgery using alizarin red staining and flow cytometry. The results showed a significant decrease in Ca^2+^ levels (Figure 4e–f), and adalimumab did not significantly improve this change.

### 3.5 Disruption of Ca^2+^ Channel Regulatory Proteins Impairs Muscle Function After Stroke

**FIGURE 5:**
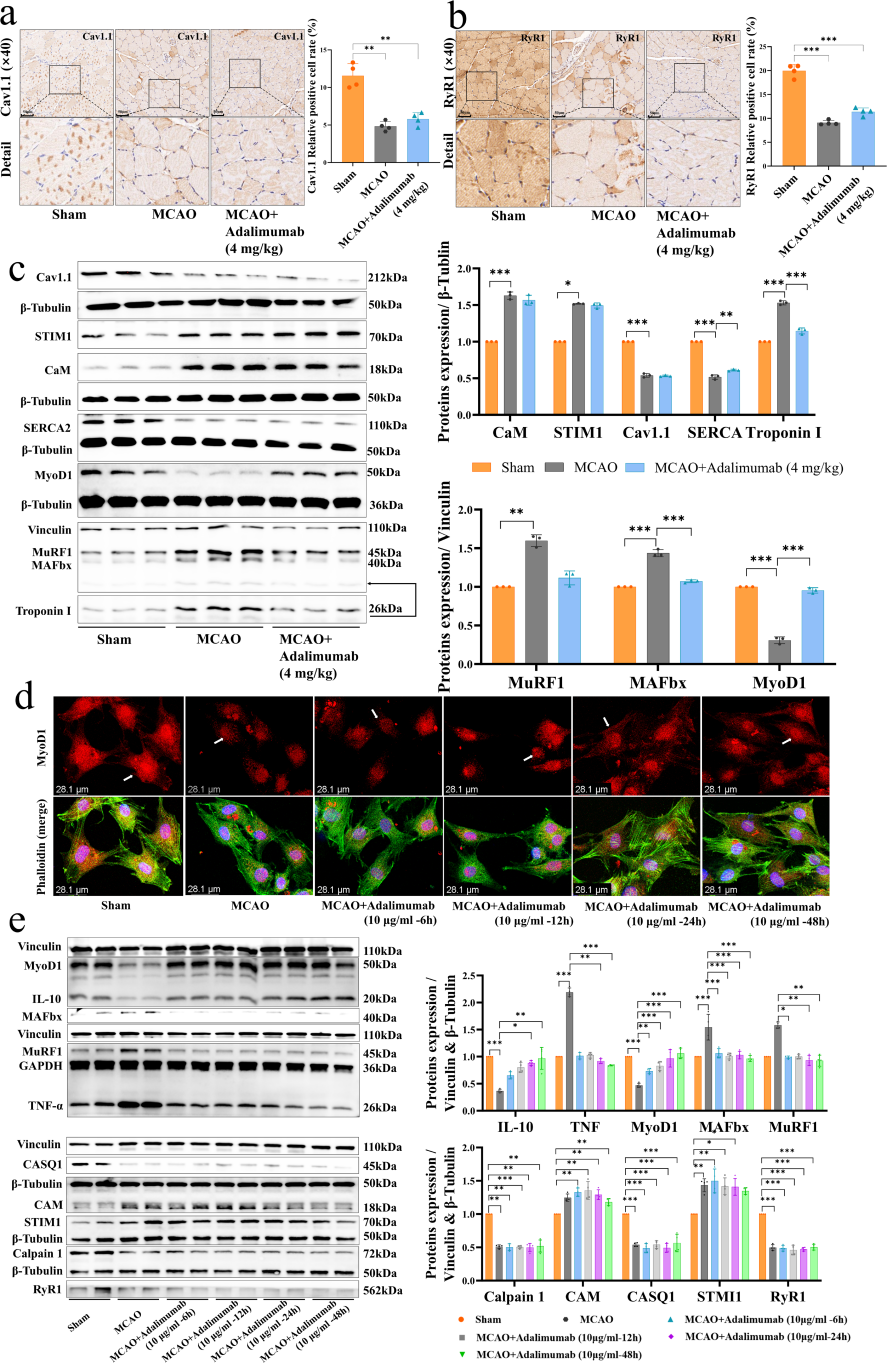
Altered Ca^2+^ channel modulatory proteins lead to poststroke muscle impairment. (a–b) IHC staining and quantification of Cav1.1 and RyR1 expression in the soleus muscle, scale bar = 50 μm, *n* = 4. (c) WB bands and quantitative analysis of Ca^2+^‐regulatory and muscle atrophy‐related proteins, including CaM, STIM1, Cav1.1, SERCA2, Troponin I, MyoD1, MuRF1 and MAFbx, *n* = 3. (d) Representative IF images showing MyoD1 and phalloidin expression at different time points following adalimumab treatment, scale bar = 28.1 μm. (e) WB bands and corresponding quantitative analysis of TNF‐α, IL‐10, MyoD1, MuRF1, MAFbx, CaM, STIM1, CASQ1, RyR1 and calpain 1 in primary muscle cells, *n* = 4. *p < 0.05, **p < 0.01, ***p < 0.001.

To investigate Ca^2+^ channel function, we examined key channel molecules. IHC and WB experiments further revealed a significant decrease in the expression of excitation–contraction coupling regulatory proteins SERCA2, Cav1.1 and RYR1, while Ca^2+^ sensing proteins STIM1 and CAM showed compensatory upregulation (Figure 5a–c). These results indicate impaired contraction–relaxation in MCAO rats, with disrupted Ca^2+^ homeostasis likely contributing to muscle strength loss. To investigate the time‐dependent effects of adalimumab, we treated MCAO rat soleus muscle primary cells with adalimumab (10 μg/mL) for different durations. Immunofluorescence analysis (IF) results showed that with prolonged adalimumab treatment, the expression levels of MyoD1 and cytoskeletal‐related proteins increased (Figure 5d), while the expression of muscle atrophy factors TNF‐α, MAFbx and MuRF1 significantly decreased. Compared with MCAO, adalimumab did not significantly alter CASQ1, CAM, STIM1, Calpain1 or RyR1 expression (Figure 5e). These results suggest that adalimumab does not regulate the expression of calcium‐regulatory proteins closely associated with muscle strength recovery.

### 3.6 Combined Adalimumab and GV‐58 Therapy Restores Intracellular and Mitochondrial Ca^2+^ Levels

**FIGURE 6:**
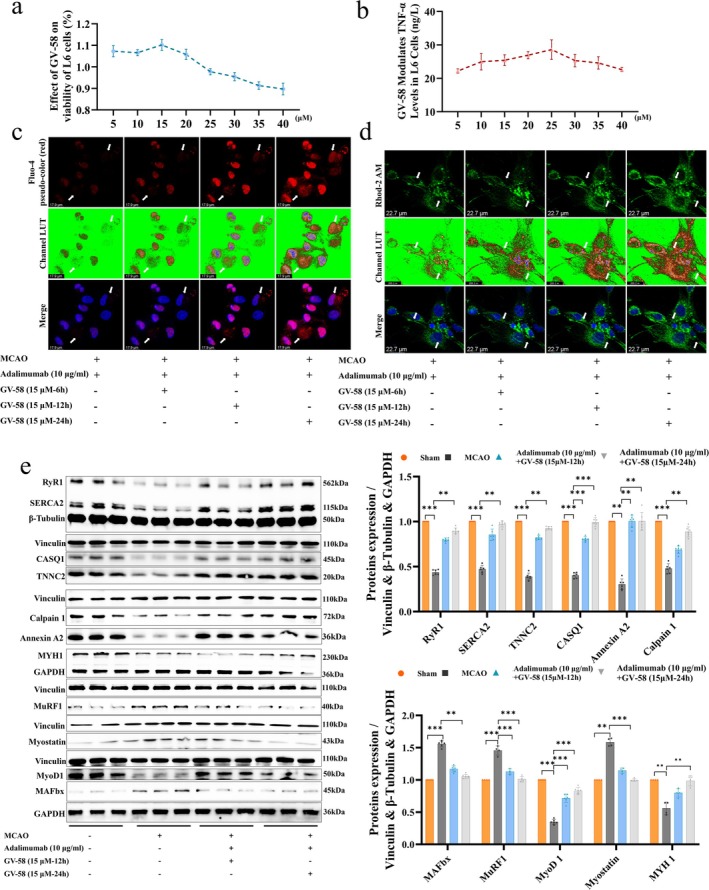
Synergistic treatment with adalimumab and Gv‐58 activates Ca^2+^ regulatory pathways and suppresses muscle atrophy markers. (a) Cell viability analysis following GV‐58 treatment. (b) Effects of GV‐58 on TNF‐α expression levels in primary cells. (c–d) Time‐dependent IF images showing changes in cytosolic and mitochondrial Ca^2+^ levels after GV‐58 and adalimumab co‐treatment, scale bar = 17.9 μm, scale bar = 22.7 μm. (e) Representative WB bands and expression analysis of RyR1, SERCA2, TNNC2, CASQ1, Annexin A2, calpain 1, MyoD1, MuRF1, MAFbx, Myostatin and MYH1 in response to combination therapy at different time points, *n* = 6. *p < 0.05, **p < 0.01, ***p < 0.001.

After confirming that the tool drug GV‐58 showed no significant toxicity to the MCAO primary muscle cells (Figure 6a–b), we treated primary muscle cells with a combination of adalimumab and GV‐58 (15 μM) and continuously monitored the Ca^2+^ concentrations both intracellularly and within the mitochondria. With increasing treatment time, the Ca^2+^ concentrations in both the MCAO primary muscle cells and mitochondria significantly increased (Figure 6c). Compared to the MCAO group, the combined treatment group showed a significant increase in the expression of key Ca^2+^ regulatory proteins, including RyR1, SERCA2, TNNC2, CASQ1, Annexin A2 and Calpain 1, while the expression of MAFbx, MuRF1 and Myostatin significantly decreased (Figure 6e). These results suggest that the recovery of muscle health in MCAO rats is primarily regulated by two mechanisms: First, the maintenance of muscle structure relies on key regulators of protein synthesis and degradation; second, the restoration of muscle strength through the functional modulation of Ca^2+^ channels and calcium storage proteins.

### 3.7 Muscle Structural and Functional Recovery Relies on Parallel Modulation of Atrophy Pathways and Calcium Homeostasis

**FIGURE 7:**
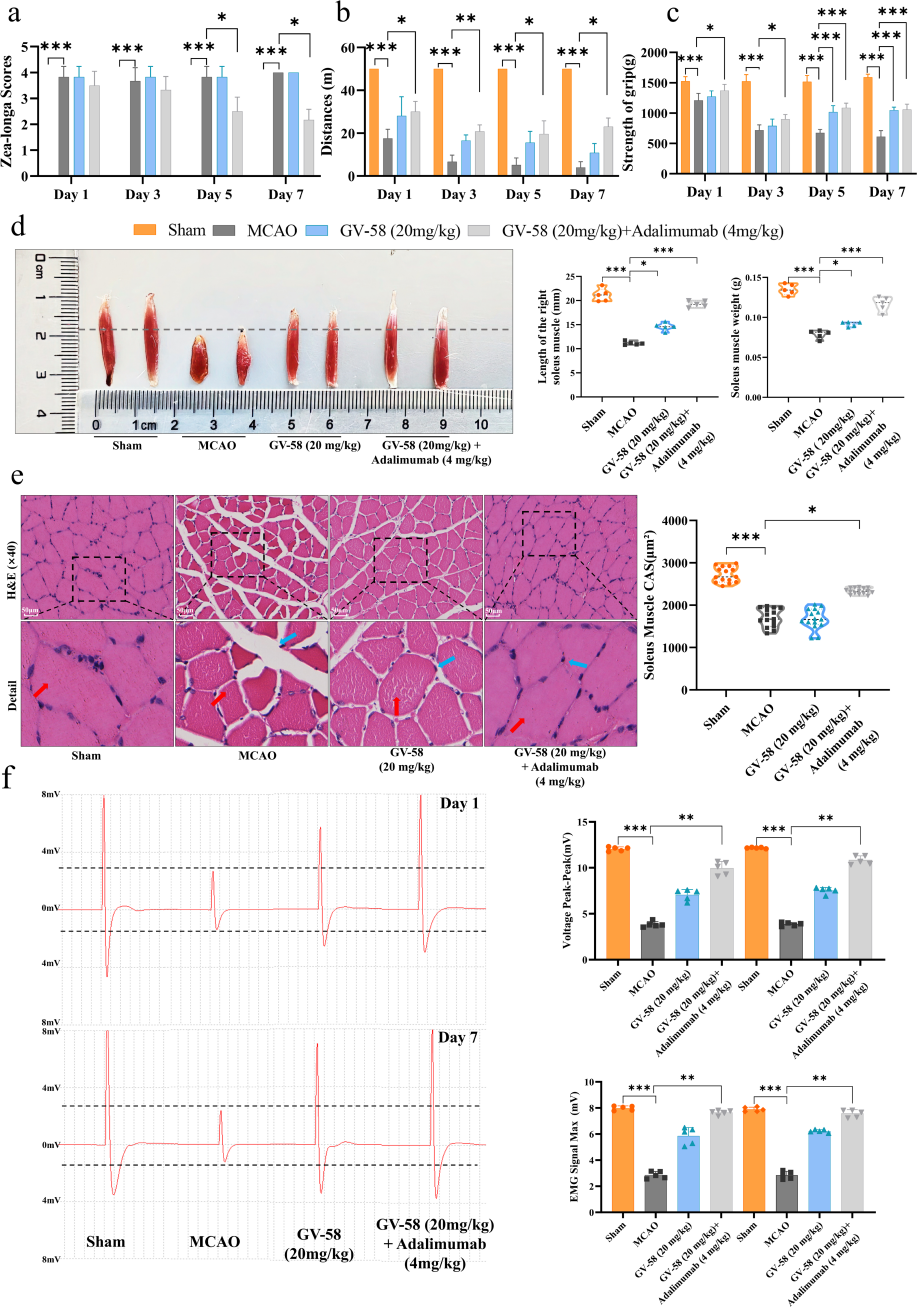
Effects of combined treatment with GV‐58 and adalimumab on muscle function and structure in MCAO rats. (a) Zea‐Longa neurological scores following combination therapy. (b–c) Quantitative analysis of locomotor distance and forelimb grip strength. (d) Representative images and quantification of soleus muscle length and weight after intervention, *n* = 5. (e) Representative H&E‐stained sections showing histological changes in soleus muscle, scale bar = 50 μm. (f) Representative images of muscle electrical signals in rats and intensity analysis, *n* = 5. *p < 0.05, **p < 0.01, ***p < 0.001.

To validate the effects of adalimumab combined with GV‐58 on muscle structure and function in ISS rats, we assessed behavioural parameters and the expression changes of key proteins. The results showed that combined treatment significantly improved neurological deficits, increased locomotor distance and enhanced forelimb grip strength (Figure 7a–c). Compared to the MCAO group, the combined treatment group showed a significant increase in the length and weight of the soleus muscle (Figure 7d). Histopathological analysis revealed a significant increase in the cross‐sectional area of muscle fibres in the combined treatment group (Figure 7e). In addition, the peak‐to‐peak value and maximum value of the electromyogram were also significantly enhanced (Figure 7f).

### 3.8 Combination Therapy Restores Calcium Homeostasis and Enhances Muscle Regeneration

**FIGURE 8:**
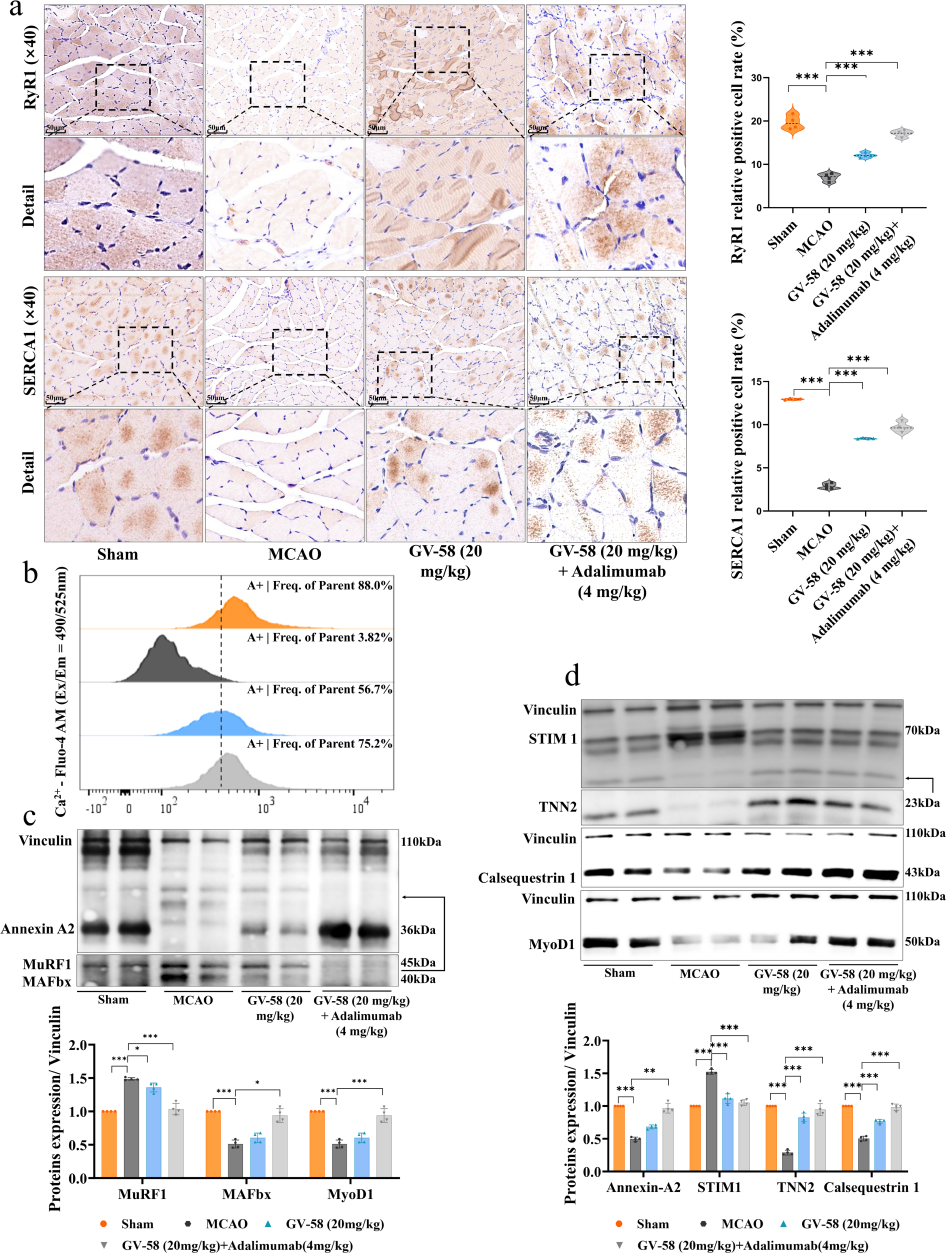
Effects of combined treatment with GV‐58 and adalimumab on muscle Ca^2+^ homeostasis in MCAO rats. (a) IHC staining and quantification of RyR1 and SERCA1 expression in soleus muscle, scale bar = 50 μm, *n* = 4. (b) FC analysis of intracellular Ca^2+^ levels in skeletal muscle. (c–d) WB detection of Annexin A2, TNN2, Calsequestrin 1, STIM1, MyoD1, MuRF1 and MAFbx expression following combination treatment, *n* = 4. *p < 0.05, **p < 0.01, ***p < 0.001.

We further investigated the effects of the combination of GV‐58 and adalimumab on Ca^2+^ and regulatory channel activity in MCAO rats. IHC revealed markedly increased expression of key Ca^2+^ regulatory proteins, RyR1 and SERCA1, in soleus muscle (Figure 8a). Flow cytometry confirmed a substantial restoration of Ca^2+^ levels in muscle tissue (Figure 8b). WB results showed an increase in the expression of Annexin A2, TNN2 and Calsequestrin 1, while the compensatory upregulation of STIM1 was significantly inhibited. Additionally, the expression of muscle atrophy markers MuRF1 and MAFbx was significantly downregulated, and the expression of myogenesis‐related factor MyoD1 was significantly upregulated (Figure 8c–d). These results indicate that the combined treatment helps restore muscle Ca^2+^ homeostasis and promotes the balance between protein degradation and synthesis processes.

## 4 Discussion

After IS, muscle atrophy is significantly pronounced due to central nervous system dysfunction [32], metabolic imbalances and tissue inflammation [33]. The stroke patients often experience significant muscle atrophy and functional impairment [34]. Studies have shown that the TNF‐α–mediated inflammatory pathway plays a central role in muscle atrophy [35]. Furthermore, previous studies have also demonstrated that inhibiting the ISS‐related tissue inflammation can significantly improve ISS and enhance motor function. Although ISS is secondary to stroke, it shares core pathological features with primary sarcopenia [36], such as the loss of muscle mass, strength and function, as defined by EWGSOP criteria [37]. In this context, studies have confirmed that adalimumab, an effective anti‐inflammatory treatment, can improve muscle atrophy and motor dysfunction related to cachexia [38]. The current study aimed to explore the therapeutic effects of adalimumab on ISS and provide a theoretical basis for developing and optimising monoclonal antibody‐based therapies.

TNF‐α signalling can regulate muscle protein turnover under inflammatory conditions. This study investigated the therapeutic potential of adalimumab in an MCAO‐induced ISS model. Mechanistically, adalimumab modulated the expression levels of MyoD1, MuRF1 and MAFbx, which are crucial regulators of muscle protein synthesis and degradation, thereby improving uscle histomorphology. Notably, despite improving histomorphology, muscle strength remained compromised, suggesting a dissociation between morphological recovery and contractile functionality. This phenomenon is also observed in clinical settings [39, 40]. For instance, imaging or muscle biopsy during poststroke rehabilitation showed that some patients exhibited restored muscle fibre alignment and fascicular integrity; however, their motor performance remained significantly impaired [41, 42]. This suggests that unctional recovery might rely more on complex neuromuscular coordination and the proper functioning of ion channels [43].

To elucidate the mechanisms underlying persistent muscle weakness in ISS, transcriptomic analysis revealed a significant increase in the calcium signalling pathways–related genes, highlighting potential deficits in excitation–contraction coupling. Functional analyses confirmed that the MCAO rats exhibited impaired calcium handling, including reduced calcium storage and suppressed expression of key Ca^2+^ channel proteins (Cav1.1, RYR1), which were not restored by adalimumab treatment. Further in vitro validation revealed that adalimumab could effectively mitigate inflammation and muscle protein degradation; however, it could not rescue the expression of essential calcium regulatory proteins, such as CASQ1, STIM1 and CAM. These findings suggested that calcium dysregulation might constitute a critical bottleneck, limiting muscle force recovery. In order to overcome this barrier, a combination strategy using adalimumab and the calcium channel agonist GV‐58 was implemented. GV‐58 is a potent Cav1.1 agonist that enhances calcium influx by prolonging the open state of voltage‐dependent calcium channels, thereby improving the excitation–contraction coupling efficiency in denervated muscle fibres. This dual‐target intervention restored calcium channel and metabolism‐related protein expression, significantly improving behaviour and muscle pathology.

Notably, the combined administration of adalimumab and GV‐58 restored muscle structure and function to a certain extent; however, there remains room for further improvement. The combined strategy depended on the availability of sufficient baseline energy to exert its full therapeutic potential. Conceptually, the human body functions as a highly sophisticated biological system that requires not only the repair of damaged components but also adequate metabolic ‘fuel’ to resume optimal performance [44]. This interpretation aligns with the accumulating clinical evidence emphasizing the importance of energy intake and nutritional support in the functional recovery of poststroke patients.

In conclusion, this study introduced a novel paradigm of ‘anti‐inflammatory and calcium channel activation’ dual‐target therapy for the treatment of ISS. This combined treatment strategy deepened our understanding of the complex pathological mechanisms of poststroke sarcopenia and provided new insights and potential translational potential for the precise intervention in sarcopenia secondary to neuroinflammation or neurodegenerative diseases. A limitation of this study was the use of RNA‐seq, which cannot distinguish cell type‐specific transcriptional alterations. Future studies using single‐cell RNA‐seq or spatial transcriptomics might help clarify the cellular targets of adalimumab and GV‐58 within the muscle microenvironment. Future studies should further explore the long‐term clinical effects of these combined interventions and their generalizability across diverse stroke models.

## 5 Conclusions

This study demonstrates that coordinated targeting of TNF‐α–mediated inflammation with adalimumab and calcium channel dysfunction with GV‐58 is essential for restoring both muscle architecture and contractile function in poststroke sarcopenia, underscoring the therapeutic potential of dual‐modality intervention.

## Disclosure

All human and animal studies have been approved by the appropriate ethics committee and have therefore been performed in accordance with the ethical standards laid down in the 1964 Declaration of Helsinki and its later amendments.

## Ethics Statement

This protocol was approved by the Laboratory Animal Ethics Committee of Chengdu University of Traditional Chinese Medicine (No. 2024054).

## Conflicts of Interest

The authors declare no conflicts of interest.

## Supporting information


**Fig. S1** **Adalimumab has no significant effect on healthy muscle.** (a) Representative images of laser speckle in rat brain, scale bar = 1 mm. (b) Muscle electrical signals in rats and intensity analysis, *n* = 5. (c) Soleus muscle from each group and quantification of soleus muscle length and weight, *n* = 5. (d) H&E staining of soleus muscle showing morphological alterations and IHC results of MuRF1 in soleus muscle, scale bar = 50 μm, *n* = 5. (e) WB bands and quantitative analysis of MyoD1, MuRF1, MAFbx, RyR1, TNNC2, CASQ1 in soleus muscle, *n* = 4. ns: no significance.

**Data S1:** Supplementary information.

**Table S1:** Detailed reagent information.
**Table S2:** Abbreviations.
**Table S3:** Instruments and equipment.

## Data Availability

The data are available from the corresponding author on reasonable request.
