# Supplementary figures and images for: Muscle Structure and Function Recovery: Adalimumab‐Calcium Channel Synergy in Post–Ischemic Stroke Sarcopenia
Source: J Cachexia Sarcopenia Muscle. 2025 Nov 10;16(6):e70097. doi: 10.1002/jcsm.70097 (PMC12598305; doi:10.1002/jcsm.70097)

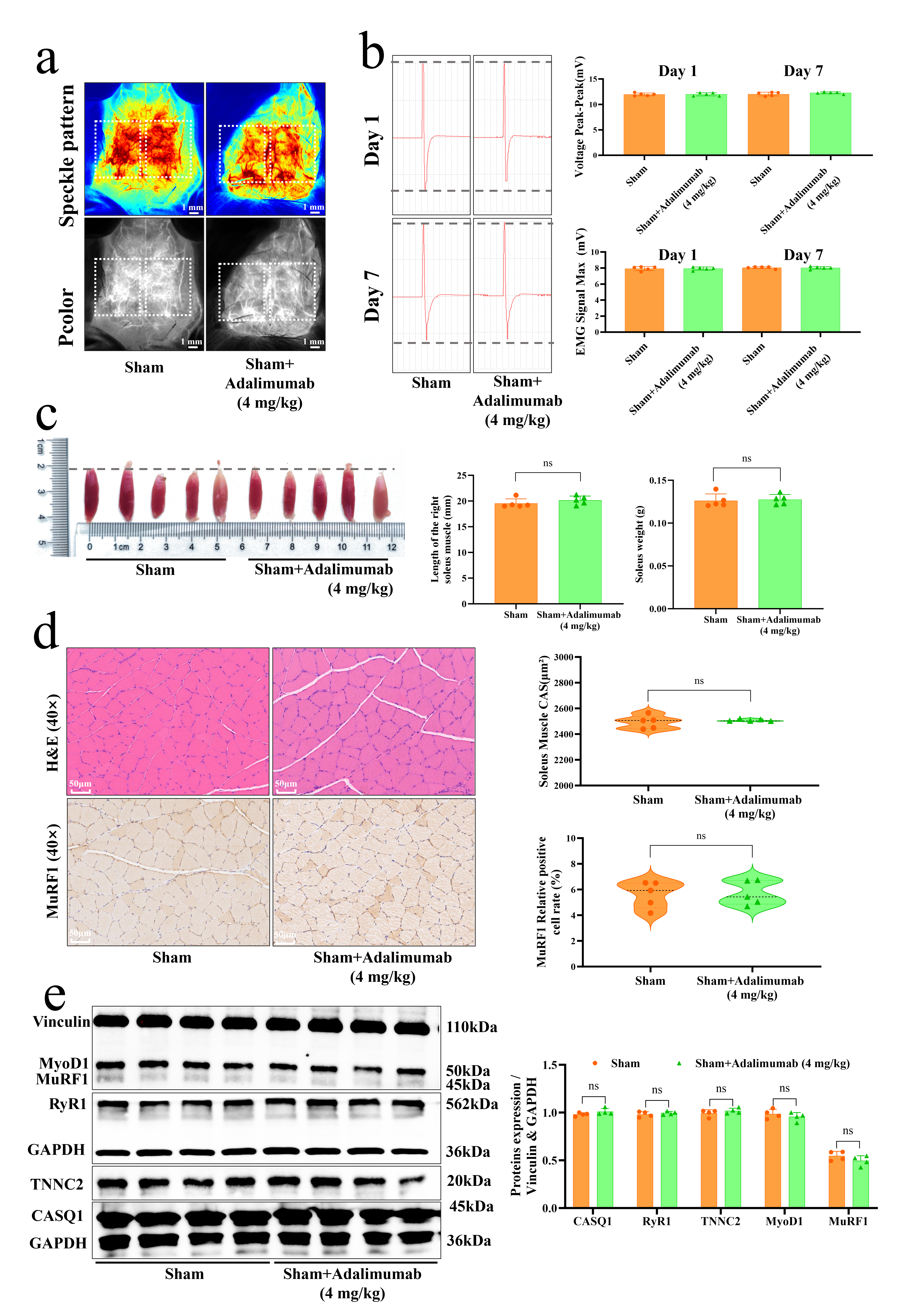

Supplement: Supplementary file 1 — Fig. S1 Adalimumab has no significant effect on healthy muscle. (a) Representative images of laser speckle in rat brain, scale bar = 1 mm. (b) Muscle electrical signals in rats and intensity analysis, n = 5. (c) Soleus muscle from each group and quantification of soleus muscle length and weight, n = 5. (d) H&E staining of soleus muscle showing morphological alterations and IHC results of MuRF1 in soleus muscle, scale bar = 50 μm, n = 5. (e) WB bands and quantitative analysis of MyoD1, MuRF1, MAFbx, RyR1, TNNC2, CASQ1 in soleus muscle, n = 4. ns: no significance. [file JCSM-16-e70097-s003.tif]
