# Supplementary material for: Muscle Structure and Function Recovery: Adalimumab‐Calcium Channel Synergy in Post–Ischemic Stroke Sarcopenia
Source: J Cachexia Sarcopenia Muscle. 2025 Nov 10;16(6):e70097. doi: 10.1002/jcsm.70097 (PMC12598305; doi:10.1002/jcsm.70097)
Supplement: Supplementary file 3 — Table S1: Detailed reagent information. Table S2: Abbreviations. Table S3: Instruments and equipment. [file JCSM-16-e70097-s001.docx]

**Supplementary Table 1 Detailed Reagent Information**

| **Name** | **Company** | **Lot number** |
| --- | --- | --- |
| **Adalimumab** | MCE | HY-P9908 |
| **Adalimumab injection** | SinoCelltech Ltd. | C1202309006A |
| **Annexin A2** | Proteintech | 11256-1-AP |
| **A/G PLUS-Agarose beads** | Beyotime | P2055 |
| **BSA** | BioFroxx | 4240GR100 |
| **BCA** | Sino Biological Inc | KIT-BCA01 |
| **β-Tubulin** | Servicebio | GB12139 |
| **Calpain 1** | Abclonal | A8710 |
| **CaM** | Proteintech | 10541-1-AP |
| **CASQ1** | Abcam | ab191564 |
| **Cav1.1** | Abcam | ab2862 |
| **CCK8** | Biosharp | BS350B |
| **Cell Complete Lysis Buffer for Western and IP** | Beyotime | P0037 |
| **Cy3-labeled Goat Anti-Rabbit IgG (H+L)** | Beyotime | A0516 |
| **DMEM medium** | Gibco | C11995500BT |
| **ECL Immunoblotting Substrate kit** | 4A Biotech Co., Ltd, | 4AW011-100 |
| **FBS** | Gibco | 10091148 |
| **Fluo-4 AM** | Beyotime | S1060 |
| **GAPDH** | Servicebio | GAPDH |
| **GV-58** | Selleck | E0137 |
| **HRP Conjugated Goat Anti-rabbit IgG (H+L)** | Boster | BA1054 |
| **IL-1β detector kit** | Jiangsu Meimian Industrial Co., Ltd., | MM-0047R2 |
| **IL-6 detector kit** | Jiangsu Meimian Industrial Co., Ltd., | MM-0190R2 |
| **IL-10** | Abclonal | A2171 |
| **MAFbx** | Proteintech | 67172-1-Ig |
| **MuRF1** | Proteintech | 55456-1-AP |
| **MYH1** | Abclonal | A6935 |
| **MyoD1** | Abcam | ab307805 |
| **Myostatin** | Abcam | ab203076 |
| **Rhod-2 AM** | Beyotime | S1062S |
| **ROS detection kit** | Beyotime | S0033M |
| **RyR1** | Abcam | ab219798 |
| **SERCA** | Abcam | ab150435 |
| **Skim milk powder** | BioFroxx | 1172GR500 |
| **STIM1** | Proteintech | 81156-1-RR |
| **TNF-α** | Proteintech | 60291-1-Ig |
| **TNF-α detector kit** | Jiangsu Meimian Industrial Co., Ltd., | MM-0180R2 |
| **TNNC2** | Abclonal | A7740 |
| **Triton X-100(Reagent grade)** | Beyotime | ST1723 |
| **Troponin I** | Abclonal | A21246 |
| **TTC** | KESHI | 2024061101 |
| **Vinculin** | CST | #4650S |
| **Zoletil-50** | Virbac | BN8LXLA |
| **2% Alizarin Red S** | Beyotime | C0138 |
| **2.5% glutaraldehyde** | Biosharp | BL911A |

**Supplementary Table 2. Abbreviations**

| **Name** | **Company** |
| --- | --- |
| **Akt** | Protein Kinase B |
| **ANOVA** | Analysis of Variance |
| **AS** | Ankylosing spondylitis |
| **BSA** | Bovine Serum Albumin |
| **CAM** | Calmodulin |
| **Cav1.1** | Calcium Voltage-gated Channel Subunit Alpha1 S |
| **CASQ1** | Calsequestrin 1 |
| **CCA** | Common Carotid Artery |
| **CETSA** | Cellular Thermal Shift Assay |
| **CO₂** | Carbon Dioxide |
| **Co-IP** | Co-immunoprecipitation |
| **DAB** | Diaminobenzidine |
| **DAVID** | Database for Annotation, Visualization, and Integrated Discovery |
| **DMEM** | Dulbecco's Modified Eagle Medium |
| **ECA** | External Carotid Artery |
| **ECL** | Enhanced Chemiluminescence |
| **FBS** | Fetal Bovine Serum |
| **FC** | Flow Cytometry |
| **Fluo-4 AM** | Fluo-4 Acetoxymethyl Ester |
| **GAPDH** | Glyceraldehyde-3-phosphate dehydrogenase |
| **H&E** | Hematoxylin And Eosin |
| **HRP** | Horseradish Peroxidase |
| **ICA** | Internal Carotid Artery |
| **IF** | Immunofluorescence |
| **IHC** | Immunohistochemical |
| **IL-1β** | Interleukin-1 beta |
| **IL-6** | Interleukin-6 |
| **IL-10** | Interleukin-10 |
| **IP** | Immunoprecipitation |
| **IS** | Ischemic Stroke |
| **ISS** | Ischemic stroke-induced sarcopenia |
| **MAFbx** | Muscle Atrophy F-Box Protein |
| **MAPK** | Mitogen-Activated Protein Kinase |
| **MCAO** | Middle Cerebral Artery Occlusion |
| **mRNA** | Messenger RNA |
| **mTOR** | Mammalian Target of Rapamycin |
| **MuRF1** | Muscle RING-finger Protein-1 |
| **MYH** | Myosin Heavy Chain |
| **MYOD1** | Myoblast Determination Protein 1 |
| **NF-κB** | Nuclear Factor kappa-light-chain-enhancer of activated B cells |
| **PBS** | Phosphate buffered saline |
| **RNA** | Ribonucleic Acid |
| **ROI** | Region Of Interest |
| **ROS** | Reactive Oxygen Species |
| **RYR1** | Ryanodine Receptor 1 |
| **SDS** | Sodium Dodecyl Sulfate |
| **SERCA1** | Sarcoplasmic/Endoplasmic Reticulum Calcium ATPase 1 |
| **SERCA2** | Sarcoplasmic/Endoplasmic Reticulum Calcium ATPase 2 |
| **SPF** | Specific pathogen-free |
| **SPSS** | Statistical Product and Service Solutions |
| **STIM1** | Stromal Interaction Molecule 1 |
| **TEM** | Transmission electron microscopy |
| **TNF-α** | Tumor necrosis factor-alpha |
| **TNFR1/2** | Tumor Necrosis Factor Receptor 1/2 |
| **TNN2** | Troponin T Type 2 |
| **TNNC2** | Troponin C Type 2 (Fast Skeletal Muscle) |
| **Troponin I** | Cardiac Troponin I |
| **TTC** | 2,3,5-Triphenyltetrazolium Chloride |
| **WB** | Western Blot |

**Supplementary Table 3. Instruments and equipment**

| **Name** | **Company** | **Instrument model** |
| --- | --- | --- |
| **Constant temperature incubator** | Thermo Scientific | 3111 |
| **Scientific Centrifuges and Rotors** | Thermo Scientific | ST16R |
| **Thermostatic water bath** | Shanghai Shenshun Biological Technology Co., Ltd | W201 |
| **Horizontal shaker** | Servicebio | DS-2H200 |
| **Vortex Mixers** | hanghai Medical University Instruments Co., Ltd. | XW-80A |
| **Ultrasonic cleaner** | Kun Shan Instruments Co., Ltd | KQ-400KDE |
| **Ultrapure water meter** | ULUPURE | UPK-I-10T |
| **Barnstead** | Millipore | SYNA00000 |
| **Electronic balance** | METTLER TOLEDO | ME204E |
| **Electrophoresis power supply** | Beijing Liuyi Instrument Plant | DYY-6C |
| **High Speed Tissue Grinder** | Servicebio | KZ-11 |
| **Microplate reader** | Thermo Scientific | Varioska |
| **Vsualizer** | BIORAD | ChemiDoc |
| **Fluorescence microscope** | Leica | ICX41RFL |
| **Laser confocal microscope** | Leica | TSC SP8 |
| **Pathology Slice System** | Leica | TP1020/CM1950 |
| **flow cytometer** | BD | FACSCanto Ⅱ |
| **Ultra-high resolution system** | Leica | TSC SP8 STEP |
| **laser speckle imaging system** | RWD | ZW/RFLSI III |
| **Grip strength meter** | Zhongke Biotechnology Co., Ltd | SA417 |
| **Physiological signal system** | Chengdu Instrument Factory | RM6240XC |
| **Ultrasound system** | Vevo | 3100 LT |
